# Rapid epidemic expansion of the SARS-CoV-2 Omicron variant in southern Africa

**DOI:** 10.1038/s41586-022-04411-y

**Published:** 2022-01-07

**Authors:** Raquel Viana, Sikhulile Moyo, Daniel G. Amoako, Houriiyah Tegally, Cathrine Scheepers, Christian L. Althaus, Ugochukwu J. Anyaneji, Phillip A. Bester, Maciej F. Boni, Mohammed Chand, Wonderful T. Choga, Rachel Colquhoun, Michaela Davids, Koen Deforche, Deelan Doolabh, Louis du Plessis, Susan Engelbrecht, Josie Everatt, Jennifer Giandhari, Marta Giovanetti, Diana Hardie, Verity Hill, Nei-Yuan Hsiao, Arash Iranzadeh, Arshad Ismail, Charity Joseph, Rageema Joseph, Legodile Koopile, Sergei L. Kosakovsky Pond, Moritz U. G. Kraemer, Lesego Kuate-Lere, Oluwakemi Laguda-Akingba, Onalethatha Lesetedi-Mafoko, Richard J. Lessells, Shahin Lockman, Alexander G. Lucaci, Arisha Maharaj, Boitshoko Mahlangu, Tongai Maponga, Kamela Mahlakwane, Zinhle Makatini, Gert Marais, Dorcas Maruapula, Kereng Masupu, Mogomotsi Matshaba, Simnikiwe Mayaphi, Nokuzola Mbhele, Mpaphi B. Mbulawa, Adriano Mendes, Koleka Mlisana, Anele Mnguni, Thabo Mohale, Monika Moir, Kgomotso Moruisi, Mosepele Mosepele, Gerald Motsatsi, Modisa S. Motswaledi, Thongbotho Mphoyakgosi, Nokukhanya Msomi, Peter N. Mwangi, Yeshnee Naidoo, Noxolo Ntuli, Martin Nyaga, Lucier Olubayo, Sureshnee Pillay, Botshelo Radibe, Yajna Ramphal, Upasana Ramphal, James E. San, Lesley Scott, Roger Shapiro, Lavanya Singh, Pamela Smith-Lawrence, Wendy Stevens, Amy Strydom, Kathleen Subramoney, Naume Tebeila, Derek Tshiabuila, Joseph Tsui, Stephanie van Wyk, Steven Weaver, Constantinos K. Wibmer, Eduan Wilkinson, Nicole Wolter, Alexander E. Zarebski, Boitumelo Zuze, Dominique Goedhals, Wolfgang Preiser, Florette Treurnicht, Marietje Venter, Carolyn Williamson, Oliver G. Pybus, Jinal Bhiman, Allison Glass, Darren P. Martin, Andrew Rambaut, Simani Gaseitsiwe, Anne von Gottberg, Tulio de Oliveira

**Affiliations:** 1 Lancet Laboratories, Johannesburg, South Africa; 2 Botswana Harvard AIDS Institute Partnership, Botswana Harvard HIV Reference Laboratory, Gaborone, Botswana; 3 Harvard T.H. Chan School of Public Health, Boston, MA USA; 4 Botswana Presidential COVID-19 Taskforce, Gaborone, Botswana; 5 National Institute for Communicable Diseases (NICD) of the National Health Laboratory Service (NHLS), Johannesburg, South Africa; 6 KwaZulu-Natal Research Innovation and Sequencing Platform (KRISP), Nelson R. Mandela School of Medicine, University of KwaZulu-Natal, Durban, South Africa; 7 South African Medical Research Council Antibody Immunity Research Unit, School of Pathology, Faculty of Health Sciences, University of the Witwatersrand, Johannesburg, South Africa; 8 Institute of Social and Preventive Medicine, University of Bern, Bern, Switzerland; 9 Division of Virology, National Health Laboratory Service, Bloemfontein, South Africa; 10 Division of Virology, University of the Free State, Bloemfontein, South Africa; 11 Center for Infectious Disease Dynamics, Department of Biology, Pennsylvania State University, University Park, PA USA; 12 Diagnofirm Medical Laboratories, Gaborone, Botswana; 13 Institute of Evolutionary Biology, University of Edinburgh, Edinburgh, UK; 14 Zoonotic Arbo and Respiratory Virus Program, Centre for Viral Zoonoses, Department of Medical Virology, University of Pretoria, Pretoria, South Africa; 15 Emweb, Herent, Belgium; 16 Division of Medical Virology, Faculty of Health Sciences, University of Cape Town, Cape Town, South Africa; 17 Department of Zoology, University of Oxford, Oxford, UK; 18 Department of Biosystems Science and Engineering, ETH Zurich, Zurich, Switzerland; 19 Division of Medical Virology, Faculty of Medicine and Health Sciences, Stellenbosch University, Tygerberg, Cape Town, South Africa; 20 Laboratorio de Flavivirus, Fundacao Oswaldo Cruz, Rio de Janeiro, Brazil; 21 Laboratório de Genética Celular e Molecular, Universidade Federal de Minas Gerais, Belo Horizonte, Brazil; 22 Division of Virology, NHLS Groote Schuur Laboratory, Cape Town, South Africa; 23 Wellcome Centre for Infectious Diseases Research in Africa (CIDRI-Africa), Cape Town, South Africa; 24 Division of Computational Biology, Faculty of Health Sciences, University of Cape Town, Cape Town, South Africa; 25 Institute for Genomics and Evolutionary Medicine, Department of Biology, Temple University, Philadelphia, PA USA; 26 Health Services Management, Ministry of Health and Wellness, Gaborone, Botswana; 27 NHLS Port Elizabeth Laboratory, Port Elizabeth, South Africa; 28 Faculty of Health Sciences, Walter Sisulu University, Mthatha, South Africa; 29 Public Health Department, Integrated Disease Surveillance and Response, Ministry of Health and Wellness, Gaborone, Botswana; 30 Department of Immunology and Infectious Diseases, Harvard T.H. Chan School of Public Health, Boston, MA, USA; 31 NHLS Tygerberg Laboratory, Tygerberg Hospital, Cape Town, South Africa; 32 Department of Virology, Charlotte Maxeke Johannesburg Academic Hospital, Johannesburg, South Africa; 33 Botswana-Baylor Children’s Clinical Centre of Excellence, Gaborone, Botswana; 34 Baylor College of Medicine, Houston, TX USA; 35 Department of Medical Virology, University of Pretoria, Pretoria, South Africa; 36 National Health Laboratory, Health Services Management, Ministry of Health and Wellness, Gaborone, Botswana; 37 National Health Laboratory Service (NHLS), Johannesburg, South Africa; 38 Centre for the AIDS Programme of Research in South Africa (CAPRISA), Durban, South Africa; 39 Centre for Epidemic Response and Innovation (CERI), School of Data Science and Computational Thinking, Stellenbosch University, Stellenbosch, South Africa; 40 Department of Medicine, Faculty of Medicine, University of Botswana, Gaborone, Botswana; 41 Department of Medical Laboratory Sciences, School of Allied Health Professions, Faculty of Health Sciences, University of Botswana, Gaborone, Botswana; 42 Discipline of Virology, School of Laboratory Medicine and Medical Sciences and National Health Laboratory Service (NHLS), University of KwaZulu-Natal, Durban, South Africa; 43 Next Generation Sequencing Unit, Division of Virology, Faculty of Health Sciences, University of the Free State, Bloemfontein, South Africa; 44 Department of Molecular Medicine and Haematology, University of the Witwatersrand, Johannesburg, South Africa; 45 School of Pathology, Faculty of Health Sciences, University of the Witwatersrand, Johannesburg, South Africa; 46 PathCare Vermaak, Pretoria, South Africa; 47 Institute of Infectious Disease and Molecular Medicine, University of Cape Town, Cape Town, South Africa; 48 Department of Molecular Pathology, School of Pathology, Faculty of Health Sciences, University of the Witwatersrand, Johannesburg, South Africa; 49 Department of Global Health, University of Washington, Seattle, WA USA

**Keywords:** Molecular evolution, Epidemiology, SARS-CoV-2

## Abstract

The SARS-CoV-2 epidemic in southern Africa has been characterized by three distinct waves. The first was associated with a mix of SARS-CoV-2 lineages, while the second and third waves were driven by the Beta (B.1.351) and Delta (B.1.617.2) variants, respectively^1–3^. In November 2021, genomic surveillance teams in South Africa and Botswana detected a new SARS-CoV-2 variant associated with a rapid resurgence of infections in Gauteng province, South Africa. Within three days of the first genome being uploaded, it was designated a variant of concern (Omicron, B.1.1.529) by the World Health Organization and, within three weeks, had been identified in 87 countries. The Omicron variant is exceptional for carrying over 30 mutations in the spike glycoprotein, which are predicted to influence antibody neutralization and spike function^4^. Here we describe the genomic profile and early transmission dynamics of Omicron, highlighting the rapid spread in regions with high levels of population immunity.

## Abstract (web summary)

The genomic profile and early transmission dynamics of the Omicron strain of SARS-CoV-2.

## Main

Since the onset of the COVID-19 pandemic in December 2019, variants of SARS-CoV-2 have emerged repeatedly. Some variants have spread worldwide and made major contributions to the cyclical infection waves that occur asynchronously in different regions. Between October and December 2020, the world witnessed the emergence of the first variants of concern (VOCs). These variants exhibited increased transmissibility and/or immune evasion properties that threatened global efforts to control the pandemic. Although the Alpha (B.1.1.7), Beta and Gamma VOCs^2,5^ that emerged during this time disseminated globally and drove epidemic resurgences in many different countries, it was the highly transmissible Delta variant that subsequently displaced all of the other VOCs in most regions of the world^6^. During its spread, the Delta variant evolved into multiple sublineages^7^, some of which demonstrated signs of having a growth advantage in certain locations^8^, prompting speculation that the next VOC to drive a resurgence of infections would probably be derived from Delta. In October 2021, while Delta was continuing to exhibit high levels of transmission in the Northern Hemisphere, a large Delta wave was subsiding in southern Africa. The culmination of this wave coincided with the emergence of a new SARS-CoV-2 variant that, within days of its near-simultaneous discovery in four individuals in Botswana, a traveller from South Africa in Hong Kong and 54 individuals in South Africa, was designated by the World Health Organization (WHO) as Omicron—the fifth VOC of SARS-CoV-2. Since then and the beginning of 2022, over 100,000 genomes of Omicron have been produced as Omicron has started to dominate SARS-CoV-2 infections in the world.

## Epidemic dynamics and detection of Omicron

**Fig. 1:**
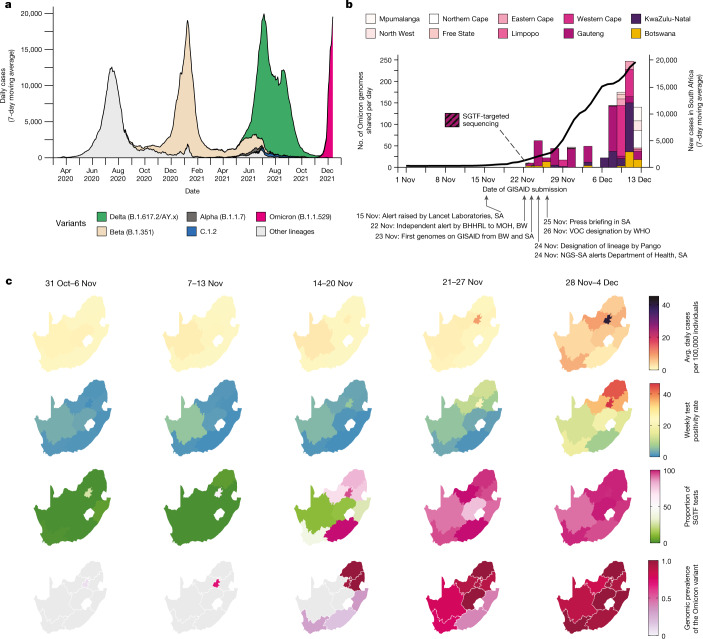
Detection of Omicron variant. **a**, The progression of daily reported cases in South Africa from March 2020 to December 2021. The 7-day rolling average of daily case numbers is coloured by the inferred proportion of variants responsible for the infections, as calculated by genomic surveillance data on GISAID. **b**, Timeline of Omicron detection in Botswana and South Africa. Bars represent the number of Omicron genomes shared per day, according to the date they were uploaded to GISAID; the line represents the 7-day moving average of daily new cases in South Africa. BHHRL, Botswana Harvard HIV Reference Laboratory; BW, Botswana; NGS-SA, Network for Genomic Surveillance in South Africa; SA, South Africa. **c**, Weekly progression of average daily cases per 100,000 individuals, test positivity rates, proportion of SGTF tests (on the TaqPath COVID-19 PCR assay) and genomic prevalence of Omicron in nine provinces of South Africa for five weeks from 31 October to 4 December 2021. Note that, because of heterogeneous use of the TaqPath PCR assay across provinces, the proportion of SGTF tests illustrated for the Eastern Cape province in weeks of 14–20 November and 21–27 November 2021 are based on only 2 and 4 data points, respectively. Genomic prevalence here is equivalent to the proportion of weekly surveillance sequences genotyped as being Omicron.

The three distinct epidemic waves of SARS-CoV-2 experienced by southern African countries were each driven by different variants: the first between June and August 2020 by descendants of the B.1 lineage^1^; the second between November 2020 and February 2021 by the Beta VOC^2,9^; and the third between May and September 2021 by the Delta VOC^3^, with an estimated 2–5% of third-wave cases in South Africa attributed to the C.1.2 lineage^10^ (Fig. 1a). Serosurveys conducted before the Delta wave suggested high levels of exposure to SARS-CoV-2 (40–60%) in South Africa^11,12^, and the estimated seroprevalence was >70% in Gauteng on the basis of a population-based survey that was conducted between October and December 2021 (ref. ^13^). The weeks following the third wave in South Africa, between 10 October and 15 November 2021, were marked by lower levels of transmission, as indicated by a low incidence of reported COVID-19 cases (100–200 new cases per day) and low (<2%) test positivity rates (Fig. 1a–c).

A rapid increase in COVID-19 cases was observed from the middle of November 2021 in Gauteng province, the economic hub of South Africa containing the cities of Tshwane (Pretoria) and Johannesburg. Specifically, rising case numbers and test positivity rates were first noticed in Tshwane, initially associated with outbreaks in higher-education settings. This resurgence of cases was accompanied by an increasing frequency of *S*-gene target failure (SGTF) during TaqPath-based diagnostic PCR testing: a phenomenon that was previously observed with the Alpha variant due to a deletion at amino acid positions 69 and 70 (∆69–70) in the SARS-CoV-2 spike protein^14^. Given the low prevalence of Alpha in South Africa (Fig. 1a), targeted whole-genome sequencing of these specimens was prioritized.

**Extended Data Fig. 1:**
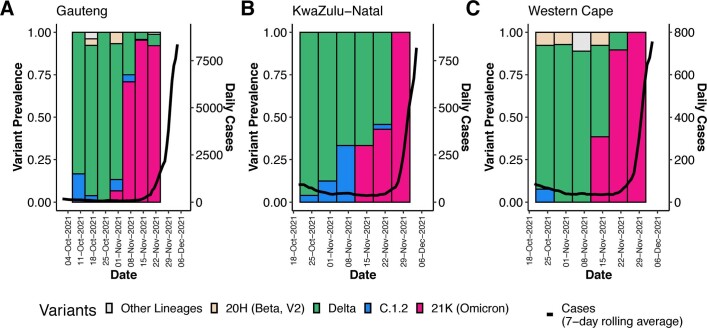
Progression of daily recorded cases and variant proportions in Gauteng (A), KwaZulu-Natal (B) and Western Cape (C) provinces between October and December 2021. A sharp increase in the 7-day rolling average of the number of cases is observed in all three of the biggest provinces in South Africa at the emergence of the Omicron variant.

On 19 November 2021, sequencing results from a batch of 8 SGTF samples collected between 14 and 16 November 2021 indicated that all were of a new and genetically distinct lineage of SARS-CoV-2. Further rapid sequencing identified the same variant in 29 out of 32 routine diagnostic samples from multiple locations in Gauteng province, indicating the widespread circulation of this new variant by the second week of November. Crucially, this rise immediately preceded a sharp increase in reported case numbers (Fig. 1c, Extended Data Fig. 1). In the following four days, the presence of this lineage was confirmed by sequencing in another two provinces—KwaZulu-Natal and the Western Cape (Fig. 1b).

Concurrently, in Gaborone, Botswana (<360 km from Tshwane), four genomes generated from samples collected on 11 November 2021 and sequenced on 17–18 November 2021 as part of weekly surveillance displayed an unusual set of mutations. These were reported to the Botswana Ministry of Health and Wellness on 22 November 2021 as unusual sequences that were linked to a group of visitors (non-residents) on a diplomatic mission. The sequences were uploaded to GISAID^15,16^ on 23 November 2021, and it became apparent that they belonged to a new lineage. A further 15 genomically confirmed cases (not epidemiologically linked to the first four) were identified within the same week from various other locations in Botswana. All of these either had travel links from South Africa, or were contacts of someone with travel links.

**Extended Data Fig. 2:**
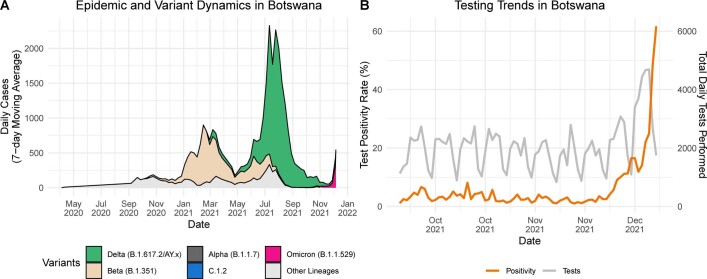
Epidemic Progression in Botswana. **A**) Epidemic and variant dynamics in Botswana from May 2020 to December 2021, with the 7-day rolling average of the number of recorded cases coloured by the proportion of variants as inferred by genomic surveillance data available on GISAID. At the end of November 2021, a big Delta-driven wave was coming to its end and an Omicron wave was starting at the end of November 2021. **B**) Trends in testing numbers and positivity rates in Botswana between October and December 2021, showing a sharp increase in positivity rate mid-November 2021.

On 24 November 2021, these SARS-CoV-2 genomes from both South Africa and Botswana were designated as belonging to a new PANGO lineage (B.1.1.529)^17^, which was later divided into sublineages aliased BA.1 (the main clade), BA.2 and BA.3. On 26 November 2021, the lineage was designated a VOC and named Omicron by the WHO on the recommendation of the Technical Advisory Group on SARS-CoV-2 Virus Evolution^18^. By the first week of December 2021, Omicron was causing a rapid and sustained increase in cases in South Africa and Botswana (Fig. 1c, Extended Data Fig. 2 (for Botswana)). In Gauteng, weekly test positivity rates increased from <1% in the week beginning 31 October, to 16% in the week beginning 21 November 2021, and to 35% in the week beginning 28 November, concurrent with an exponential rise in COVID-19 incidence (Fig. 1c, Extended Data Fig. 1). Nationally, daily case numbers exceeded 22,000 (84% of the peak of the previous wave of infections) by 9 December 2021. At the same time, the proportion of TaqPath PCR tests with SGTF increased rapidly in all provinces of South Africa, reaching ~90% nationally by the week beginning 21 November 2021, strongly indicating that the fourth wave was being driven by Omicron—an indication that has now been confirmed by virus genome sequencing in all provinces (Fig. 1c). Similarly, Botswana experienced a sharp increase in cases, doubling every 2–3 days during late November to early December 2021, transitioning from a 7-day moving average of <10 cases per 100,000 individuals to above 25 cases per 100,000 individuals in less than 10 days (Extended Data Fig. 2).

**Extended Data Fig. 3:**
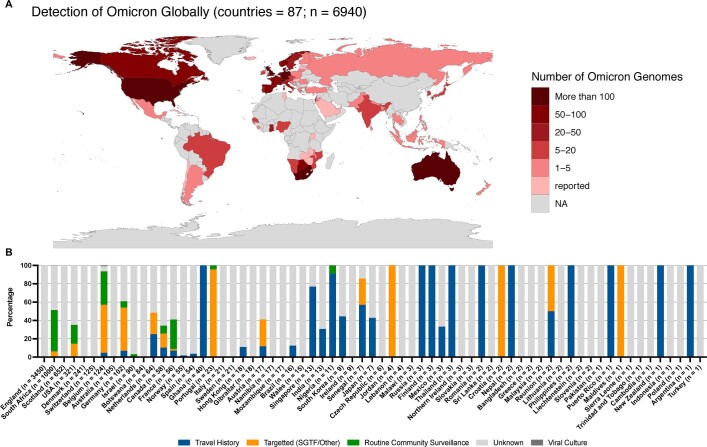
Global distribution of Omicron. (**A**) Detection of Omicron globally. Shown are the locations for which Omicron genomes have been deposited on GISAID as of December 16, 2021. Those labelled as “reported” referred to the country from which Omicron has been reported to the WHO but there is currently no sequencing data available in GISAID, all data comes from GISAID and the WHO weekly epidemiology report Edition 70 dated December 14, 2021 (https://reliefweb.int/sites/reliefweb.int/files/resources/20211207_Weekly_Epi_Update_69-%281%29.pdf). Countries are coloured according to the number of genomes deposited with warmer colours representing more genomes. (**B**) Omicron transmission globally. Shown are countries for which Omicron sequencing data is available on GISAID. Proportions of sequences are coloured according to sampling strategy or additional host/location information from either travel history, targeted sequencing (specifically for SGTF, vaccine breakthroughs, outbreaks, contact tracing or other reasons), routine surveillance or unknown if no information has been provided. Countries are ordered by the number of sequences available on GISAID as of December 16, 2021.

By 16 December 2021, Omicron had been detected in 87 countries, both in samples from travellers returning from southern Africa, and in samples from routine community testing (Extended Data Fig. 3) and, by 1 January 2022, over 100,000 genomes had been produced from over 100 countries and Omicron was becoming the dominant VOC in the world.

## Evolutionary origins of Omicron

**Fig. 2:**
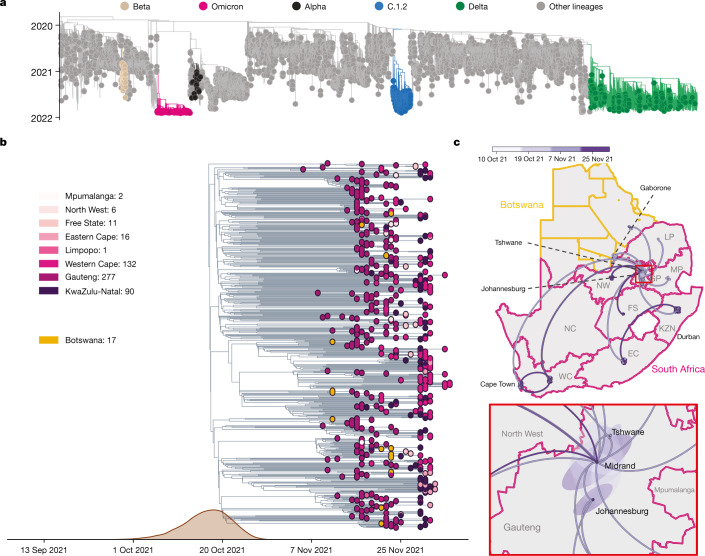
Evolution of Omicron. **a**, Time-resolved maximum likelihood phylogeny of 13,295 SARS-CoV-2 genomes; 9,944 of these are from Africa (denoted with tip point circle shapes). Alpha, Beta and Delta VOCs and the C.1.2 lineage, recently circulating in South Africa, are denoted in black, brown, green and blue, respectively. The newly identified SARS-CoV-2 Omicron variant is shown in pink. Genomes of other lineages are shown in grey. **b**, Time-resolved maximum clade credibility phylogeny of the Omicron cluster of southern African genomes (*n* = 553), with locations indicated. The posterior distribution of the TMRCA is also shown. **c**, Spatiotemporal reconstruction of the spread of the Omicron variant in southern Africa with an inset of Gauteng province. Circles represent nodes of the maximum clade credibility phylogeny, coloured according to their inferred time of occurrence (scale in the top panel). Shaded areas represent the 80% HPD interval and depict the uncertainty of the phylogeographical estimates for each node. Solid curved lines denote the links between nodes and the directionality of movement is anticlockwise along the curve. EC, Eastern Cape; FS, Free State; GP, Gauteng; KZN, KwaZulu-Natal; LP, Limpopo; MP, Mpumalanga; NC, Northern Cape; NW, North West; WC, Western Cape.

**Extended Data Fig. 4:**
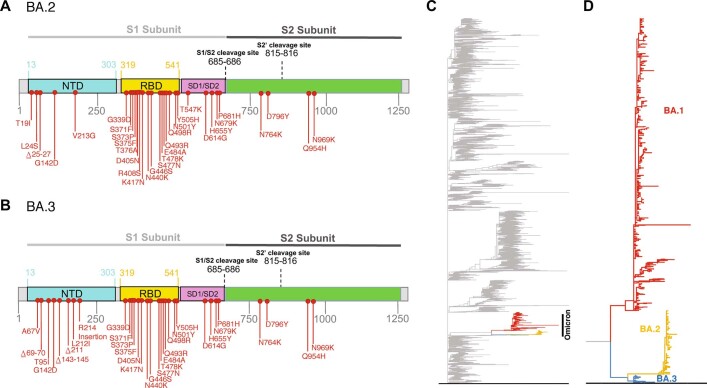
Related Lineages BA.2 and BA.3 Molecular Profile and Evolutionary Origins. **A**) Amino-acid mutations on the spike gene of the BA.2 **B**) Amino-acid mutations on the spike gene of the BA.3 **C**) Raw maximum likelihood phylogeny of 13,462 SARS-CoV-2 genomes, including 148 BA.2 and 19 BA.3. The newly identified SARS-CoV-2 Omicron variant is shown in colour versus grey for all other lineages. **D**) A zoomed-in view of the Omicron clade showing the evolutionary relationship between BA.1, BA.2 and BA.3.

To determine when and where Omicron probably originated, we analysed all 686 available Omicron genomes (including 248 from southern Africa and 438 from elsewhere in the world) retrieved from GISAID (date of access, 7 December 2021)^15,16^, in the context of a global reference set of representative SARS-CoV-2 genomes (*n* = 12,609) collected between December 2019 and November 2021. Preliminary maximum-likelihood phylogenies identified the Omicron BA.1 sequences as a monophyletic clade rooted within the B.1.1 lineage (Nextstrain clade 20B), with no clear basal progenitor (Fig. 2a). Importantly, the BA.1 cluster is highly phylogenetically distinct from any known VOCs or variants of interest (VOIs) and from any other lineages that are known to be circulating in southern Africa (such as C.1.2) (Fig. 2a). More recently, two related lineages have emerged (BA.2 and BA.3), both sharing many, but not all of the characteristic mutations of BA.1 and both having many unique mutations of their own (Extended Data Fig. 4a, b). While BA.2 and BA.3 are evolutionarily linked to BA.1 in that they all branch off of the same B.1.1 node without obvious progenitors, the three sublineages evolved independently from one another along separate branches (Extended Data Fig. 4c, d). The earliest specimens of BA.2 and BA.3 were both sampled after the earliest known BA.1 in South Africa (8 November 2021 at the time of writing), on 17 November 2021 in Tshwane (Gauteng) and on 18 November 2021 in a neighbouring province (North West), respectively. We primarily focus here on the BA.1 lineage, which is rapidly spreading in multiple countries around the world and is the lineage that was first officially designated as the Omicron VOC.

Time-calibrated Bayesian phylogenetic analysis of all BA.1 assigned genomes from southern Africa (as of 11 December 2021, *n* = 553) estimated the time at which the most recent common ancestor (TMRCA) of the analysed BA.1 lineage sequences existed to be 9 October 2021 (95% highest posterior density (HPD) 30 September–20 October) with a per-day exponential growth rate of 0.137 (95% HPD = 0.099–0.175) reflecting a doubling time of 5.1 days (95% HPD = 4.0–7.0) (Fig. 2b). These estimates are robust to whether the evolutionary rate is estimated from the data or fixed to previously estimated values (Extended Data Table 1). Limiting the analysis to genomes from Gauteng province only yields a faster growth rate estimate with a doubling time of 2.8 days (95% HPD = 2.1–4.2) (Extended Data Table 1). Using a phylodynamic model that accounts for variable genome sampling through time (birth–death skyline model (BDSKY)^19^) yields a doubling time of BA.1-assigned genomes from South Africa and Botswana (*n* = 552) of 3.9 (95% HPD = 3.5–4.3) days with an effective reproduction number (*R*_e_) of 2.79 (95% HPD = 2.60–2.97) during the period from early November to early December. The BDSKY-estimated *R*_e_ for the Gauteng province dataset is 3.86 (95% HPD = 3.43–4.29) and 3.61 (95% HPD = 3.20–4.02) for the 3-epoch and 4-epoch model, respectively (Extended Data Tables 4 and 5). Spatiotemporal phylogeographic analysis indicates that the BA.1 variant spread from the Gauteng province of South Africa to seven of the eight other provinces and to two regions of Botswana from late October to late November 2021, and shows evidence of more recent transmission within and between other South African provinces (Fig. 2c). However, this does not imply that Omicron originated in Gauteng and these phylogeographic inferences could change as further genomic data accumulate from other locations.

## Molecular profile of Omicron

**Fig. 3:**
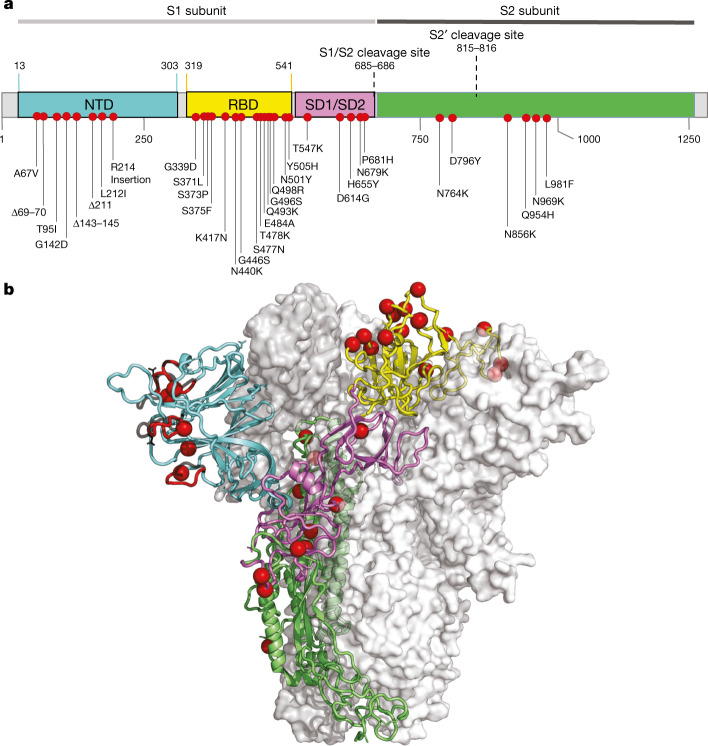
Molecular profile of BA.1. **a**, Amino acid mutations on the spike gene of the BA.1 variant. **b**, The structure of the SARS-CoV-2 spike trimer, showing a single spike protomer in cartoon view. The NTD, RBD, subdomains 1 and 2, and the S2 protein are shown in cyan, yellow, pink, and green, respectively. The red spheres indicate the alpha carbon positions for each omicron variant residue. NTD-specific loop insertions/deletions are shown in red, with the original loop shown in transparent black.

Compared with Wuhan-Hu-1, BA.1 carries 15 mutations in the spike receptor-binding domain (RBD) (Fig. 3), five of which (G339D, N440K, S477N, T478K and N501Y) have been shown individually to enhance bind to human ACE2 (hACE2)^20^. Seven of the RBD mutations (K417N, G446S, E484A, Q493R, G496S, Q498R and N501Y) are expected to have moderate to strong effects on the binding of at least three out of the four major classes of RBD-targeted neutralizing antibodies^21–23^. These RBD mutations coupled with four amino acid substitutions (A67V, T95I, G142D and L212I), three deletions (69–70, 143–145 and 211) and an insertion (EPE between 214 and 215) in the N-terminal domain (NTD)^24^ are predicted to underlie the substantially reduced sensitivity of Omicron to neutralization by anti-SARS-CoV-2 antibodies induced by either infection or vaccination^25,26^. These mutations also involve key structural epitopes that are targeted by some of the currently authorized monoclonal antibodies, particularly bamlanivimab + etesevimab and casirivimab + imdevimab^26–29^. Preliminary analysis suggests that, although the spike mutations involve a number of T cell and B cell epitopes, the majority of epitopes (>70%) remain unaffected^30^.

**Extended Data Fig. 5:**
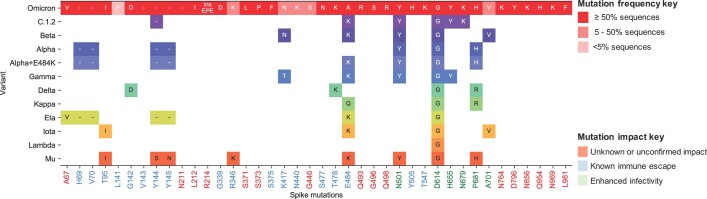
BA.1 spike mutations shared with other VOC/VOIs. All spike mutations seen in BA.1 are listed at the top in red and coloured according to prevalence. Prevalence was calculated by number of mutation detections / total number of sequences. However, primer drop-outs have affected the RBD region spanning K417N, N440K and G446S, and so it is likely that these mutations may actually be more prevalent than indicated here. For the VOC/VOIs only mutations that are shared with Omicron and seen in ≥50% of the respective VOC/VOI sequences are shown and are coloured according to Nextstrain clade. The mutations listed at the bottom are shaded according to known immune escape (blue), enhanced infectivity (green) or for unknown/unconfirmed impact (red).

Omicron also has a cluster of three mutations (H655Y, N679K and P681H) adjacent to the S1/S2 furin cleavage site (FCS) that are likely to enhance spike protein cleavage and fusion with host cells^31,32^ and that could also contribute to enhanced transmissibility^33^ (Extended Data Fig. 5).

Outside of the spike protein, a deletion in nsp6 (del105–107), in the same region as deletions seen in Alpha, Beta, Gamma and Lambda, may have a role in evasion of innate immunity^34^, and the double mutation in nucleocapsid (R203K and G204R)—which is also present in Alpha, Gamma and C.1.2—has been associated with enhanced infectivity in human lung cells^35^.

## Recombination analysis

Given the large number of mutations differentiating BA.1, BA.2 and BA.3 from other known SARS-CoV-2 lineages, it was considered plausible that (1) all of these lineages might have descended from a common recombinant ancestor; (2) one or more of the BA lineages might have originated through recombination between a virus in one of the other BA lineages and a virus in a non-BA lineage; or (3) one of the BA lineages may have originated through recombination between viruses in the other two BA lineages. We tested these hypotheses using a variety of recombination detection approaches (implemented using GARD^36^, 3SEQ^37^ and RDP5 (ref. ^38^)) to identify potential signals of recombination in sequence datasets containing the BA.1, BA.2 and BA.3 sequences together with sequences representative of global SARS-CoV-2 genomic diversity.

**Extended Data Fig. 6:**
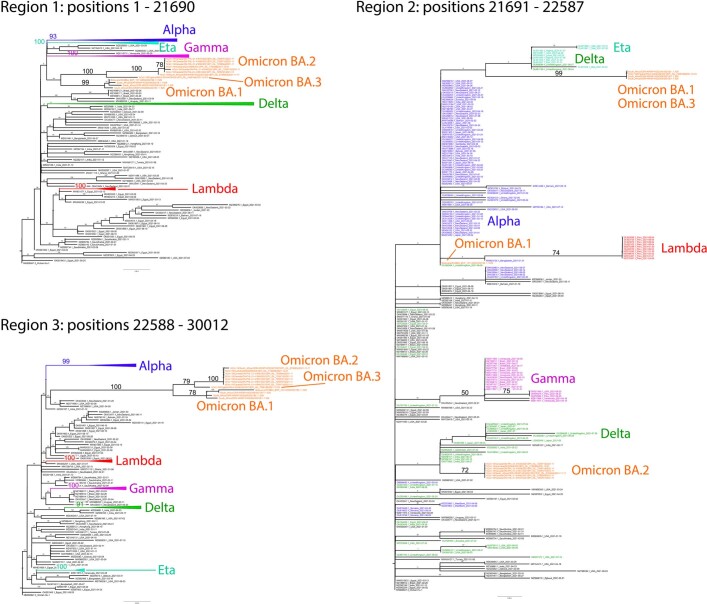
Maximum-likelihood trees (inferred with RAxML v8.2.12^82^) for genome regions bounding the consensus recombination breakpoints detected in lineages BA.1, BA.2 and BA.3^83^. The trees include SARS-CoV-2 genome sequences sampled in 2021 (N = 221) together with 13 sequences representing the BA.1, BA.2 and BA.3 lineages. Whereas in trees for regions 1 and 3 BA.2 and BA.3 cluster together with high bootstrap support, BA.1 is a well-supported albeit more distantly related sibling lineage. The a 897nt region 2 segment (encoding the N-terminal domain of spike) includes 67 polymorphic sites with a maximum 8nt difference between strains, showing little bootstrap support for any sibling or clade relationships except the membership of certain viruses in WHO-designated clades (Lambda, Omicron, Gamma). Despite Omicron lineages BA.1 and BA.3 clustering with certain Delta and Eta viruses and Omicron BA.2 clustering with a distinct set of Delta viruses (all on the basis of several key nucleotide positions), trees based on region 2 show no statistical support for the three Omicron lineages having distinct evolutionary origins. Bootstrap values are shown on branches with relevant values magnified for readability. All trees were rooted on the Wuhan-Hu-1 sequence.

Potential evidence of a single recombination event involving BA.1, BA.2 and BA3 was identified by 3SEQ (*P* = 0.03), GARD (delta c-AIC = 20) and RDP5 (GENECONV *P* = 0.027; RDP *P* = 0.006) within the NTD encoding region of spike. The most likely breakpoint locations for this recombination event were 21690 for the 5′ breakpoint (high likelihood interval between 15716 and 21761) and 22198 for the 3′ breakpoint (high likelihood interval between 22197 and 22774). However, these analyses could not reliably identify which of BA.1, BA.2 or BA.3 was the recombinant. Phylogenetic analysis of the genome regions bounded by these breakpoints (genome coordinates 1–21689, 21690–22198 and 22199–29903) potentially supported (1) BA.1 having acquired the NTD encoding region of BA.3 through recombination, (2) BA.3 having acquired the NTD-encoding region of BA.1 through recombination or (3) BA.2 having acquired the NTD-encoding region of a non-BA lineage virus through recombination (Extended Data Fig. 6).

Although we found weak statistical and phylogenetic evidence of one of BA.1, BA.2 or BA.3 being recombinant, we found no evidence that the MRCA of the BA.1, BA.2 and BA.3 lineages was recombinant. However, note that recombination tests in general will not have sufficient statistical power to reliably identify evidence of individual recombination events that result in transfers of less than ~5 contiguous polymorphic nucleotide sites between genomes^36,39,40^. Furthermore, if BA.1, BA.2 and/or BA.3 are the products of a series of multiple partially overlapping recombination events occurring across multiple temporally clustered replication cycles, the complex patterns of nucleotide variation that might result could be extremely difficult to interpret as recombination using the methods applied here^41^.

## Selection analysis of Omicron

The large numbers of mutations seen in the BA.1, BA.2 and BA.3 lineage sequences may have accrued at an accelerated pace under the influence of positive selection. To test for evidence of this, we applied a selection analysis pipeline to all of the available sequences designated as BA.1, BA.2 and BA.3 in GISAID as of 20 December 2021. We ran selection screens individually on BA.1, BA.2 and BA.3 sequences, according to a previously described procedure^34^. We downsampled alignments of individual protein-encoding regions to obtain a median of 110 genetically unique BA.1 sequences, 3 BA.2 sequences, 2.5 BA.3 sequences and around 100 other unique sequences for each gene/open reading frame (ORF) from a representative collection of other SARS-CoV-2 lineages (used as background sequences to contextualize evolution within the Omicron subclade).

Given that the BA.1 lineage has 1,000-fold more sequences than BA.2 and BA.3, we performed the most detailed analysis on it. We detected evidence of gene-wide positive selection (using the BUSTED method^42^) acting on 11 genes or ORFs since the ancestral BA.1 lineage split from the B.1.1 lineage: *M* gene (*P* = 0.002), *N* gene (*P* = 0.006), *nsp3* (*P* = 0.05), *S* gene, exonuclease, *RdRp*, methyltransferase, helicase, *ORF7a*, *ORF6* and *ORF3a* (*P* < 0.0001 for all tests). In all ten genes, this selection was strong (ratio of non-synonymous to synonymous substitutions (d*N*/d*S*) > 5) and occurred in bursts (≤6% of branch–site combinations selected). The branch separating BA.1 from its most recent B.1.1 ancestor had the most prominent selection signal (which was strongest in the *S* gene, with evidence for nine positively selected sites along this branch^43^), strongly supporting the hypothesis that adaptive evolution had a substantial role in the mutational divergence of Omicron from other B.1.1 SARS-CoV-2 lineages. Relative to the intensity of selection evident within the background B.1.1 lineages, selection in five genes was probably significantly intensified in the BA.1 lineage: *S* gene (intensification factor *K* = 2.1, *P* < 0.0001^44^), exonuclease (*K* = 3.50, *P* = 0.0009), *nsp6* (*K* = 2.4, *P* = 0.03), *RdRp* (*K* = 1.14, *P* = 0.02) and *M* (*K* = 4.6, *P* < 0.0001).

Among 1,546 codon sites that are polymorphic among the BA.1 sequences analysed, 45 were found to have experienced episodic positive selection since BA.1 split from the B.1.1 lineage^45^ (MEME *P* ≤ 0.01; Extended Data Table 2) . Twenty-three (51%) of these codon sites are in the *S* gene, thirteen of which contain BA.1-lineage-defining mutations (that is, these selection signals reflect mutations that occurred within the ancestral Omicron lineage). The three positively selected codon sites that did not correspond to sites of lineage-defining mutations (*S*, 346; *S*, 452; and *S*, 701) are particularly notable as these are attributable to mutations that have occurred since the MRCA of the analysed BA.1 sequences. The mutations driving the positive selection signals at these three sites in the Omicron *S* gene converge on mutations seen in other VOCs or VOIs (R346K in Mu, L452R in Delta, and A701V in Beta and Iota). The A701V mutation, the precise impact of which is currently unknown, is one of 19 in a proposed ‘501Y-lineage spike meta-signature’ comprising the set of mutations that were most adaptive during the evolution of the Alpha, Beta and Gamma VOC lineages^34^. Furthermore, both R346K and L452R are known to affect antibody binding^22^ and both of the codon sites at which these mutations occur display evidence of directional selection (using the FADE method^46^). These selective patterns suggest that, during its current rapid spread, BA.1 may be undergoing additional evolution to modify its neutralization profile.

As the numbers of available BA.2 and BA.3 sequences are much lower than for BA.1, the power to perform selection detection was much reduced and not possible for some genomic regions. Nonetheless, there was a strong signal of selection on the *S* gene (*P* < 0.0001 for BA.2 and *P* = 0.05 for BA.3) and selective pressures on this gene in the BA.2 clade were intensified relative to reference SARS-CoV-2 isolates (*K* = 6.25, *P* = 0.005). Within BA.2 sequences, positive selection was detectable on five sites in the *S* gene (371, 376, 405, 477 and 505—all clade defining sites) as well on two sites in the *M* gene (19 and 63—both clade-defining sites). Within BA.3 sequences, positive selection was detectable on four sites in the *S* gene (67, 371, 477 and 505—all clade-defining sites) as well on two sites in the *N* gene (13 and 413—both clade defining sites).

## Transmissibility and immune evasion

**Fig. 4:**
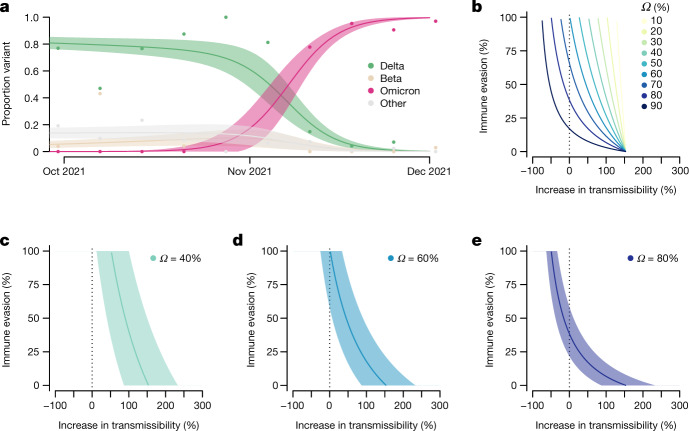
Growth of Omicron in Gauteng, South Africa, and the relationship between potential increase in transmissibility and immune evasion. **a**, Omicron rapidly outcompeted Delta in November 2021. Model fits are based on a multinomial logistic regression. Dots represent the weekly proportions of variants. **b**, The relationship between the potential increase in transmissibility and immune evasion strongly depends on the assumed level of current population immunity against Delta that is afforded by previous infections during earlier epidemic waves and/or vaccination (*Ω*). **c**–**e**, The relationship for a population immunity of 40% (**c**), 60% (**d**) and 80% (**e**) against infection and transmission with Delta. The dark vertical dashed line indicates equal transmissibility of Omicron compared to Delta. The shaded areas correspond to the 95% CIs of the model estimates.

We estimated that Omicron had a growth advantage of 0.24 (95% CI = 0.16–0.33) per day over Delta in Gauteng, South Africa (Fig. 4a). This corresponds to a 5.4-fold (95% CI = 3.1–10.1) weekly increase in cases compared with Delta. The growth advantage of Omicron is likely to be mediated by (1) an increase relative to other variants in its intrinsic transmissibility, (2) an increase relative to other variants in its ability to infect, and be transmitted from, previously infected and vaccinated individuals; or (3) both.

The predicted combination of transmissibility and immune evasion for Omicron strongly depends on the assumed level of current population immunity against infection by, and transmission of, the competing variant Delta that is afforded by previous infections with Beta, Delta and other strains during the three previous epidemic waves in South Africa, and/or vaccination (Fig. 4b). For moderate levels of population immunity against Delta (*Ω* = 0.4), immune evasion alone cannot explain the observed growth advantage of Omicron (Fig. 4c). For medium levels of immunity against Delta (*Ω* = 0.6), very high levels of immune evasion could explain the observed growth advantage without an additional increase in transmissibility (Fig. 4d). For high levels of population immunity against Delta (*Ω* = 0.8), even moderate levels of immune evasion (~25–50%) can explain the observed growth advantage without an additional increase in transmissibility (Fig. 4e). The results of seroprevalence studies and vaccination coverage (∼40% of the adult population in South Africa) suggest that the proportion of the population with potential immunity against Delta and earlier variants is probably above 60% (refs. ^11,12^). We therefore argue that the population level of protective immunity against Delta acquired during previous epidemic waves is high, and that partial immune evasion is a major driver for the observed dynamics of Omicron in South Africa. This notion is supported by recent findings that show an increased risk of SARS-CoV-2 reinfection associated with the emergence of Omicron in South Africa^47^ and the initial results from neutralization assays^48^. However, in addition to immune evasion, an increase or decrease in the transmissibility of Omicron compared with Delta cannot be ruled out.

There are a number of limitations to this analysis. First, we estimated the growth advantage of Omicron based on early sequence data only. These data could be biased due to targeted sequencing of SGTF samples and stochastic effects (such as superspreading) in a low-incidence setting, which can lead to overestimates of the growth advantage and, consequently, of the increased transmissibility and immune evasion. Second, without reliable estimates of the level of protective immunity against Delta in South Africa, we cannot obtain precise estimates of transmissibility or immune evasion of Omicron.

## Conclusion

Strong genomic surveillance systems in South Africa and Botswana enabled the identification of Omicron within a week of observing a resurgence in cases in Gauteng province. Immediate notification of the WHO and early designation as a VOC has stimulated global scientific efforts and has given other countries time to prepare their response. Omicron is now driving a fourth wave of the SARS-CoV-2 epidemic in southern Africa, and is spreading rapidly in several other countries. Genotypic and phenotypic data suggest that Omicron has the capacity for substantial evasion of neutralizing antibody responses, and modelling suggests that immune evasion could be a major driver of the observed transmission dynamics. Close monitoring of the spread of Omicron in countries outside southern Africa will be necessary to better understand its transmissibility and the capacity of this variant to evade post-infection and vaccine-elicited immunity. Neutralizing antibodies are only one component of the immune protection from vaccines and prior infection, and the cellular immune response is predicted to be less affected by the mutations in Omicron. Vaccination therefore remains critical to protect those who have the highest risk of severe disease and death. The emergence and rapid spread of Omicron poses a threat to the world and a particular threat in Africa, where fewer than one in ten people are fully vaccinated.

## Methods

### Epidemiological dynamics

We analysed daily cases of SARS-CoV-2 in South Africa up to 14 December 2021 from publicly released data provided by the National Department of Health and the National Institute for Communicable Diseases. This was accessible through the repository of the Data Science for Social Impact Research Group at the University of Pretoria (https://github.com/dsfsi/covid19za)^49,50^. The National Department of Health releases daily updates on the number of confirmed new cases, deaths and recoveries, with a breakdown by province. Daily case numbers for Botswana were obtained through Our World in Data (OWID) COVID-19 data repository (https://github.com/owid/covid-19-data). We obtained test positivity data from weekly reports from the National Institute for Communicable Diseases (NICD)^51^. Data to calculate the proportion of positive TaqPath COVID-19 PCR tests (Thermo Fisher Scientific) with SGTF in South Africa was obtained from the National Health Laboratory Service and Lancet Laboratories. Test positivity data for Botswana was obtained from the National Health Laboratory up to 6 December 2021. All data visualization was generated through the ggplot package in R^52^.

### SARS-CoV-2 sampling

As part of the NGS-SA, seven sequencing hubs in South Africa receive randomly selected samples for sequencing every week according to approved protocols at each site^53^. These samples include remnant nucleic acid extracts or remnant nasopharyngeal and oropharyngeal swab samples from routine diagnostic SARS-CoV-2 PCR testing from public and private laboratories in South Africa. In response to a focal resurgence of COVID-19 in the City of Tshwane Metropolitan Municipality in Gauteng province in November, we enriched our routine sampling with additional samples from the affected area, including initial targeted sequencing of SGTF samples. In Botswana, all public and private laboratories submit randomly selected residual nasopharyngeal and oropharyngeal PCR positive samples weekly to the National Health Laboratory (NHL) and the Botswana Harvard HIV Reference Laboratory (BHHRL) for sequencing.

### Ethical statement

The genomic surveillance in South Africa was approved by the University of KwaZulu-Natal Biomedical Research Ethics Committee (BREC/00001510/2020), the University of the Witwatersrand Human Research Ethics Committee (HREC) (M180832), Stellenbosch University HREC (N20/04/008_COVID-19), University of Cape Town HREC (383/2020), University of Pretoria HREC (H101/17) and the University of the Free State Health Sciences Research Ethics Committee (UFS-HSD2020/1860/2710). The genomic sequencing in Botswana was conducted as part of the national vaccine roll-out plan and was approved by the Health Research and Development Committee (Health Research Ethics body, HRDC#00948 and HRDC#00904). Individual participant consent was not required for the genomic surveillance. This requirement was waived by the Research Ethics Committees.

### Ion Torrent Genexus Integrated Sequencer methodology for rapid whole-genome sequencing of SARS-CoV-2

Viral RNA was extracted using the MagNA Pure 96 DNA and Viral Nucleic Acid kit on the automated MagNA Pure 96 system (Roche Diagnostics) according to the manufacturer’s instructions. Extracts were then screened by quantitative PCR to acquire the mean cycle threshold (*C*_t_) values for the SARS-CoV-2 *N* and *ORF1ab* genes using the TaqMan 2019-nCoV assay kit v1 (Thermo Fisher Scientific) on the ViiA7 Real-time PCR system (Thermo Fisher Scientific) according to the manufacturer’s instructions. Extracts were sorted into batches of *n* = 8 within a C_*t*_ range difference of 5 for a maximum of two batches per run. Extracts with <200 copies were sequenced using the low viral titre protocol. Next-generation sequencing was performed using the Ion AmpliSeq SARS-CoV-2 Research Panel on the Ion Torrent Genexus Integrated Sequencer (Thermo Fisher Scientific), which combines automated cDNA synthesis, library preparation, templating preparation and sequencing within 24 h. The Ion Ampliseq SARS-CoV-2 Research Panel consists of two primer pools targeting 237 amplicons tiled across the SARS-CoV-2 genome providing >99% coverage of the SARS-CoV-2 genome (~30 kb) and an additional five primer pairs targeting human expression controls. The SARS-CoV-2 amplicons range from 125 bp to 275 bp in length. TRINITY was used for de novo assembly and the Iterative Refinement Meta-Assembler (IRMA) was used for genome assisted assembly as well as FastQC for quality checks.

### Whole-genome sequencing and genome assembly

RNA was extracted on an automated Chemagic 360 instrument, using the CMG-1049 kit (Perkin Elmer). The RNA was stored at −80 °C before use. Libraries for whole-genome sequencing were prepared using either the Oxford Nanopore Midnight protocol with Rapid Barcoding or the Illumina COVIDseq Assay.

#### Illumina Miseq/NextSeq

For the Illumina COVIDseq assay, the libraries were prepared according to the manufacturer’s protocol. In brief, amplicons were tagmented, followed by indexing using the Nextera UD Indexes Set A. Sequencing libraries were pooled, normalized to 4 nM and denatured with 0.2 N sodium acetate. A 8 pM sample library was spiked with 1% PhiX (PhiX Control v3 adaptor-ligated library used as a control). We sequenced libraries using the 500-cycle v2 MiSeq Reagent Kit on the Illumina MiSeq instrument (Illumina). On the Illumina NextSeq 550 instrument, sequencing was performed using the Illumina COVIDSeq protocol (Illumina), an amplicon-based next-generation sequencing approach. The first-strand synthesis was performed using random hexamers primers from Illumina and the synthesized cDNA underwent two separate multiplex PCR reactions. The pooled PCR amplified products were processed for tagmentation and adapter ligation using IDT for Illumina Nextera UD Indexes. Further enrichment and clean-up was performed according to protocols provided by the manufacturer (Illumina). Pooled samples were quantified using the Qubit 3.0 or 4.0 fluorometer (Invitrogen) and the Qubit dsDNA High Sensitivity assay kit according to the manufacturer’s instructions. The fragment sizes were analysed using the TapeStation 4200 (Invitrogen). The pooled libraries were further normalized to 4 nM concentration, and 25 μl of each normalized pool containing unique index adapter sets was combined into a new tube. The final library pool was denatured and neutralized with 0.2 N sodium hydroxide and 200 mM Tris-HCl (pH 7), respectively. Sample library (1.5 pM) was spiked with 2% PhiX. Libraries were loaded onto a 300-cycle NextSeq 500/550 HighOutput Kit v2 and run on the Illumina NextSeq 550 instrument (Illumina).

#### Midnight protocol

For Oxford Nanopore sequencing, the Midnight primer kit was used as described previously^54^. cDNA synthesis was performed on the extracted RNA using the LunaScript RT mastermix (New England BioLabs) followed by gene-specific multiplex PCR using the Midnight primer pools, which produce 1,200 bp amplicons that overlap to cover the 30 kb SARS-CoV-2 genome. Amplicons from each pool were pooled and used neat for barcoding with the Oxford Nanopore Rapid Barcoding kit according to the manufacturer’s protocol. Barcoded samples were pooled and bead-purified. After the bead clean-up, the library was loaded on a prepared R9.4.1 flow-cell. A GridION X5 or MinION sequencing run was initiated using MinKNOW software with the base-call setting switched off.

#### Genome assembly

We assembled paired-end and Nanopore .fastq reads using Genome Detective v.1.132 (https://www.genomedetective.com), which was updated for the accurate assembly and variant calling of tiled primer amplicon Illumina or Oxford Nanopore reads, and the Coronavirus Typing Tool^55^. For Illumina assembly, the GATK HaploTypeCaller --min-pruning 0 argument was added to increase mutation calling sensitivity near sequencing gaps. For Nanopore, low-coverage regions with poor alignment quality (<85% variant homogeneity) near sequencing/amplicon ends were masked to be robust against primer drop-out experienced in the spike gene, and the sensitivity for detecting short inserts using a region-local global alignment of reads was increased. We also used the wf_artic (ARTIC SARS-CoV-2) pipeline as built using the Nextflow workflow framework^56^. In some instances, mutations were confirmed visually with .bam files using Geneious v.2020.1.2 (Biomatters). The reference genome used throughout the assembly process was NC_045512.2 (numbering equivalent to MN908947.3).

Raw reads from the Illumina COVIDSeq protocol were assembled using the Exatype NGS SARS-CoV-2 pipeline v.1.6.1 (https://sars-cov-2.exatype.com/). This pipeline performs quality control on reads and then maps the reads to a reference using Examap. The reference genome used throughout the assembly process was NC_045512.2 (accession number: MN908947.3).

Several of the initial Ion Torrent genomes contained a number of frameshifts, which caused unknown variant calls. Manual inspection revealed that these were probably sequencing errors resulting in mis-assembled regions (probably due to the known error profile of Ion Torrent sequencers)^57^. To resolve this, the raw reads from the IonTorrent platform were assembled using the SARSCoV2 RECoVERY (Reconstruction of Coronavirus Genomes & Rapid Analysis) pipeline implemented in the Galaxy instance ARIES (https://aries.iss.it). This pipeline fixed the observed frameshifts, confirming that they were artefacts of mis-assembly; this subsequently resolved the variant calls. The Exatype and RECoVERY pipelines each produce a consensus sequence for each sample. These consensus sequences were manually inspected and polished using Aliview v.1.27 (http://ormbunkar.se/aliview/).

All of the sequences were deposited in GISAID (https://www.gisaid.org/)^15,16^, and the GISAID accession identifiers are included in Supplementary Table 1. Raw reads for our sequences have also been deposited at the NCBI Sequence Read Archive (BioProject: PRJNA784038).

The number and position of the Omicron mutations has affected a number of primers and caused primer drop-outs across a range of sequencing protocols, especially within the RBD (https://primer-monitor.neb.com/lineages). These primer drop-outs have resulted in a number of genomes missing stretches of the RBD, and can affect estimates of mutation prevalence and the determination of the true set of lineage-defining mutations. Given this, .bam files of all initial genomes were inspected using IG Viewer to confirm mutation calls where reference calls were suspected to be from low coverage at primer dropout sites^58^.

#### Lineage classification

We used the widespread dynamic lineage classification method from the Phylogenetic Assignment of Named Global Outbreak Lineages (PANGOLIN) software suite (https://github.com/hCoV-2019/pangolin)^17^. This is aimed at identifying the most epidemiologically important lineages of SARS-CoV-2 at the time of analysis, enabling researchers to monitor the epidemic in a particular geographical region. For the Omicron variant described in this study, the corresponding PANGO lineage designation is BA.1 (lineages v.1.2.106). When first characterized, the lineage was designated B.1.1.529, but the emergence of three sibling lineages to Omicron resulted in the split into sublineages (B.1.1.529.1, B.1.1.529.2 and B.1.1.529.3, aliased as BA.1, BA.2 and BA.3). BA.1 contains all the genomes with the original mutational constellation that was designated as Omicron and, at time of writing, is the dominant sublineage.

#### Recombination testing

To test for the possibility that the Omicron lineage (including BA.1, BA.2 and BA.3) is a recombinant of other SARS-CoV-2 lineages, we used a global subsample of sequences spanning January 2021 to August 2021. Using the NCBI SARS-CoV-2 Data hub^59,60^, we constructed a dataset containing 221 sequences by randomly sampling five sequences from each month for each continent. No Oceania samples were available from July or August, and no South American sequences were available from July 2021 (ref. ^61^). These sequences were aligned together with a set of five high-quality BA.1, six BA.2 and one BA.3 sequences (representing the known diversity of these clades on 5 December 2021) using MAFFT^62^ with the default settings. Whereas 3SEQ^37^ and RDP5 (ref. ^38^) were used to analyse this dataset, a subsample of the 39 most divergent sequences from the dataset was analysed using the GARD recombination detection method^36^. As none of these recombination detection methods normally use potentially informative deletion patterns, deletions in these alignments were recoded as nucleotide substitutions (one substitution per contiguous run of deleted nucleotides). Furthermore, to minimize multiple testing issues, BA.1, BA.2 and BA.3 were tested for evidence of recombination among one another using individual sequences from each of these lineages (CERI-KRISP-K032254, EPI_ISL_7190366 and EPI_ISL_7526186, respectively) together with the Wuhan-Hu-1 sequence (which served as a reference point for rooting the four taxon phylogeny). The default program settings were used throughout for recombination analyses, with the exception of RDP5 analysis, in which sequences were treated as linear and the window sizes for the SiScan and BootScan methods (two of the seven recombination detection methods applied in RDP5) were changed to 2,000 nucleotides.

#### Selection analyses

We investigated the nature and extent of selective forces acting on BA.1, BA.2 and BA.3 genes encoding individual protein products (respectively, a median of 110, 3 and 2.5 unique BA.1, BA.2 and BA.3 sequences per protein product encoding genome region). A subset of publicly available sequences (from the Virus Pathogen Database and Analysis Resource (ViPR); https://www.viprbrc.org/) was included as background sequences to contextualize selection signals detectable within the BA.1, BA.2 and BA.3 lineages at the levels of complete protein product encoding regions, and individual codons (a median of ~100 sequences per protein coding region). Sequences were selected, quality-checked, aligned, and processed for BUSTED, RELAX, MEME, FADE, FEL and BGM selection analyses (all implemented in HyPhy v.2.5.33)^63^ using the automated RASCL pipeline as outlined previously^2,9,34^.

#### Structure modelling

We modelled the spike protein on the basis of the Protein Data Bank coordinate set 7A94, showing the first step of the spike protein trimer activation with one RBD domain in the up position, bound to the human ACE2 receptor^64^. We used Pymol (The PyMOL Molecular Graphics System, v.2.2.0) for visualization.

#### Phylogenetic analysis

All sequences on GISAID^15,16^ designated Omicron (*n* = 686; date of access: 7 December 2021) were analysed against a globally representative reference set of SARS-CoV-2 genotypes (*n* = 12,609) spanning the entire genetic diversity observed since the start of the pandemic. In brief, the reference set included: (1) all genomes from Africa assigned to PANGO lineage B.1.1 or any of its descendents, excluding those belonging to a VOC clade; (2) a representative subsampling of global data from the publicly maintained global build of Nexstrain (https://nextstrain.org/ncov/gisaid/global); and (3) the top thirty BLAST hits when querying GISAID BLAST for BA.1 and BA.2 sequences. This sampling scheme ensures that we analyse Omicron against the closest variants of the virus. Omicron and reference sequences were aligned using Nextalign^65^. A maximum-likelihood tree topology was inferred in FastTree^66^ under the following parameters: a General Time Reversible model of nucleotide substitution and a total of 100 bootstrap replicates^67^. The resulting maximum-likelihood tree topology was transformed into a time-calibrated phylogeny in which branches along the tree were scaled in calendar time using TreeTime^68^. The resulting tree was then visualized and annotated in ggtree in R^69^. Additional BA.2 (*n* = 148) and BA.3 (*n* = 19) sequences were added to the above phylogeny after review to clarify the evolutionary relationship between BA.1, BA.2 and BA.3 (Extended Data Fig. 4c, d).

#### Time-calibrated BEAST analysis

To estimate a time-scale and growth rate from the genome sequencing data, BEAST (v.1.10.4)^70,71^ was used to sample phylogenetic trees under an exponential growth coalescent model using a strict molecular clock. All BA.1-assigned genomes from South Africa and Botswana (as of 11 December 2021) were included, with some lower coverage genomes removed, leaving a total of 553 genomes. The single South African BA.2 genome (CERI-KRISP-K032307, EPI_ISL_6795834) was included to help to stabilize the root of the BA.1 clade but the exponential growth coalescent model was applied only to BA.1 (a constant population size coalescent was used for the rest of the tree). The rate of molecular evolution was estimated from the data. Two runs of 100 million iterations were compared to assess convergence, and then post-burnin samples were pooled to summarize parameter estimates.

#### Birth–death phylogenetic analysis

We analysed the full South Africa and Botswana dataset (*n* = 552, all BA.1 assigned), and the reduced dataset containing only Gauteng province genomes (*n* = 277) using the serially sampled birth–death skyline (BDSKY) model^19^, implemented in BEAST2 (v.2.5.2)^72^. To allow for changes in genomic sampling intensity shortly after the discovery of the new lineage, we allowed the sampling proportion to vary with time while keeping all other models parameters constant over the study period. The choice of prior distributions for the model parameters is summarized in Extended Data Table 3.

For each analysis, we used an HKY substitution model and a strict clock model with a fixed clock rate of 0.75 × 10^−3^ and 1.1 × 10^−3^ substitutions per site per year (s.s.y.) for the full South Africa and Botswana dataset, and Gauteng province-only dataset, respectively. To allow for comparisons with the exponential growth coalescent model, we also repeated the analyses with clock rates fixed at those estimated from the coalescent analyses (1.2 × 10^−3^ and 0.3 × 10^−3^ s.s.y.). The mean duration of infectiousness was fixed at 10 days^73,74^. The effective reproduction number, *R*_e_, was assumed to be constant through time. The sampling proportion was assumed to be 0 before the collection time of the oldest sample and allowed to change at fixed times that were approximately equidistantly spaced between the oldest sample and the most recent sample. For Markov chain Monte Carlo (MCMC) analyses of the full South Africa and Botswana dataset, the maximum clade credibility tree from the exponential growth coalescent model was used as the starting tree. We kept the tree topology fixed, estimating only internal node heights.

To assess the robustness of our estimates of *R*_e_ under different assumptions of temporal variations in the sampling proportion, we repeated the analyses with 3 instead of 4 equidistant change-time points. All of the other model parameters and priors were kept the same.

For each analysis, we ran two independent chains of 100 million MCMC steps and sampled parameters every 10,000 steps. We used Tracer (v.1.7)^75^ to evaluate MCMC convergence for each of the individual chains (effective sample size (ESS) > 200), which were then combined using LogCombiner to obtain the final posterior distribution after removing 10% of each chain as burn-in. The results were analysed using the bdskytools package in R (https://github.com/laduplessis/bdskytools).

The resulting estimates for the time of the most recent common ancestor, exponential growth rate and doubling time are summarized in Extended Data Tables 4 and 5. With fixed clock rates of 0.75 × 10^−3^ and 1.1 × 10^−3^ s.s.y. for the full South Africa and Botswana dataset and Gauteng province-only dataset, respectively, the 3-epoch and 4-epoch BDSKY models resulted in similar estimates of the effective reproduction number, *R*_e_, for both datasets: 2.74 (95% HPD = 2.56–2.92) and 2.79 (95% HPD = 2.60–2.97) for the South Africa and Botswana dataset, and 3.86 (95% HPD = 3.43–4.29) and 3.61 (95% HPD = 3.20–4.02) for the Gauteng province-only dataset. Using a faster clock rate led to more recent common ancestors and higher estimates of the effective reproduction number and growth rate.

We examined the sensitivity of our estimates to different assumptions regarding the average duration of infectiousness by repeating the analysis of the South Africa and Botswana dataset with different fixed values of the becoming non-infectious rate: 52.1 per year and 26.1 per year, which translate to an infectious period of 7 and 14 days, respectively. These values were selected as plausible bounds based on the infectious period of asymptomatic cases and the time from symptom onset to two negative RT–PCR tests^74^. The 4-epoch model was used with a fixed clock rate of 0.75 × 10^−3^ s.s.y. in these analyses. For each analysis, we ran three independent chains of 35 million MCMC steps and sampled parameters every 10,000 steps. We used Tracer (v.1.7)^75^ to evaluate MCMC convergence for each of the individual chains (ESS > 200), which were then combined using LogCombiner to obtain the final posterior distribution after removing 10% of each chain as burn-in.

The results from the sensitivity analyses showed that our estimates are largely robust to alternative assumptions about the infectious period. On doubling of the mean duration of infectiousness from 7 to 14 days, the TMRCA remained mostly the same (10 October 2021 (95% HPD = 2 October–17 October) compared with 11 October 2021 (95% HPD = 3 October–17 October), while the doubling time shifted from 4.4 (95% HPD = 3.9–5.0) days to 3.5 (95% HPD = 3.2–3.9) days. This change in the doubling time is partially explained by differing estimates of the sampling proportion. For most of the epochs, the sampling proportion increases with the doubling time to explain the same number of sequences observed in each instance, that is, if we assume a shorter average duration of infectiousness, then we infer a slower transmission of which a greater proportion of sequences has been sampled.

#### Phylogeographic analysis

MCMC analyses were run in duplicate in BEAST (v.1.10.4)^70,71^ for a total of 100 million iterations sampling every 10,000 steps in the chain. Convergence of runs was assessed in Tracer (v.1.7.1)^75^ based on high effective sample sizes (>200) and good mixing in the chains. Maximum clade credibility trees for each run were summarized in TreeAnnotator after discarding the first 10% of the chain as burn in. Finally, the spatiotemporal dispersal of Omicron was mapped using the R package seraphim^76^.

#### Estimating transmission advantage

We analysed 805 SARS-CoV-2 sequences from Gauteng, South Africa, that were uploaded to GISAID with sample collection dates from 1 September to 1 December 2021 (ref. ^15^). We used a multinomial logistic regression model to estimate the growth advantage of Omicron compared with Delta at the time point at which the proportion of Omicron reached 50% (refs. ^77,78^). We fitted the model using the multinom function of the nnet package and estimated the growth advantage using the package emmeans in R.

The difference in the net growth rates (that is, the growth advantage) between a variant (Omicron) and the wild type (Delta) can be expressed as follows:^79^

$$\rho =(1+\tau )\beta (S+{\epsilon }(1-S))-\beta S,$$

where *τ* is the increase of the intrinsic transmissibility, *ϵ* is the level of immune evasion, *β* is the transmission rate of the wild type and *S* is the proportion of the population that is susceptible to the wild type. This relationship can be algebraically solved for *τ* and *ϵ*. We further define *R*_w_ = *βSD* as the effective reproduction number of the wild-type with *D* being the generation time. *Ω* = 1 − *S* corresponds to the proportion of the population with protective immunity against infection and subsequent transmission with the wild type.

We estimated *ϵ* for different levels of *τ* and *Ω*. To propagate the uncertainty, we constructed 95% credible intervals (CIs) of the estimates from 10,000 parameter samples of *ρ*, *D* and *R*_w_. We assumed *D* to be normally distributed with a mean of 5.2 days and a s.d. of 0.8 days (ref. ^80^). We sampled from publicly available estimates of the daily *R*_w_ based on confirmed cases during the early growth phase of Omicron in South Africa (1 October–31 October 2021; range = 0.78–0.85) (https://github.com/covid-19-Re)^81^.

### Reporting summary

Further information on research design is available in the Nature Research Reporting Summary linked to this paper.

## Online content

Any methods, additional references, Nature Research reporting summaries, source data, extended data, supplementary information, acknowledgements, peer review information; details of author contributions and competing interests; and statements of data and code availability are available at 10.1038/s41586-022-04411-y.

### Supplementary information

Supplementary Table 1

Reporting Summary

Peer Review File

## Figures and Tables

**Extended Data Table 1:**
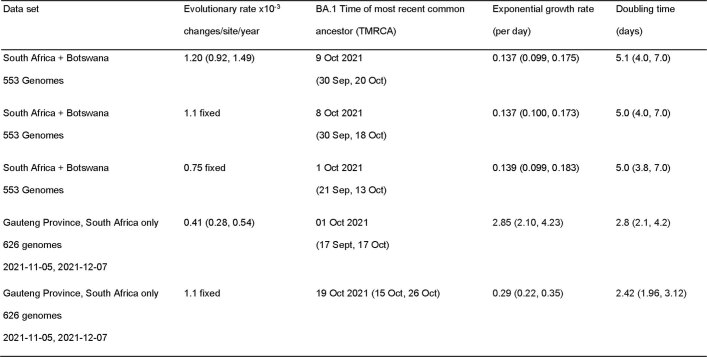
Parameter estimates from BEAST for the full South Africa and Botswana dataset and the reduced data set of only Gauteng Province genomes

95% HPD intervals in parentheses.

**Extended Data Table 2:**
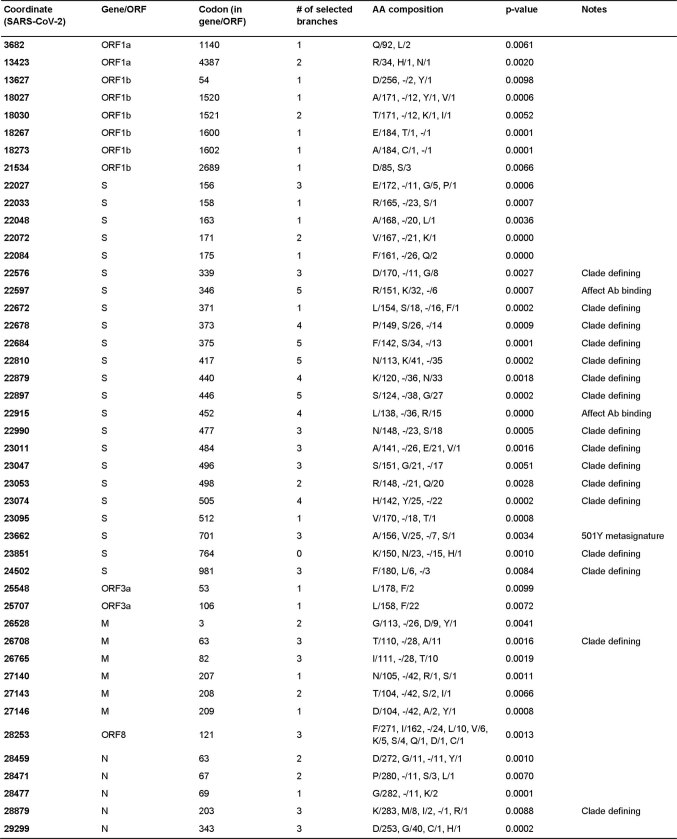
Sites in the BA.1 sequences that have been subject to episodic diversifying selection

**Extended Data Table 3:**
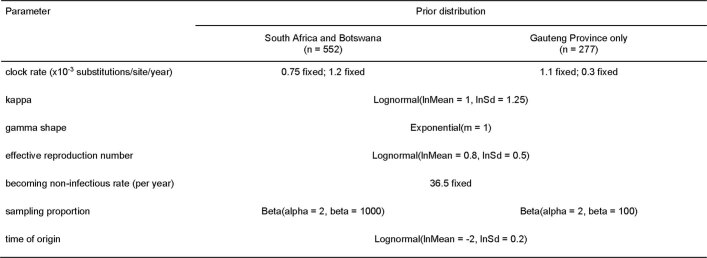
Prior distributions used for the BDSKY analyses

The becoming non-infectious rate was fixed to 36.5/year which corresponds to a mean infectious period of 10 days. A less informative prior for the sampling proportion was used for the Gauteng Province only dataset to allow for the possibility of a higher province-specific sampling proportion.

**Extended Data Table 4:**
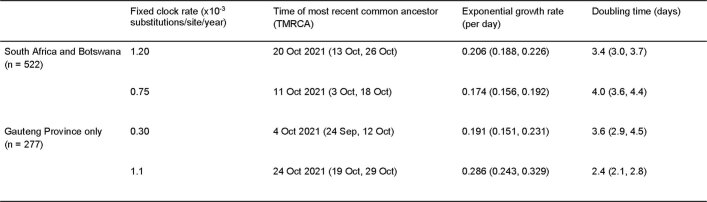
Time of most recent common ancestor, exponential growth rate and doubling time estimates for the full South Africa and Botswana dataset and the reduced dataset of only Gauteng Province genomes under the 3-epoch BDSKY model in which the sampling proportion was allowed to change at 3 equidistantly spaced time points

95% HPD intervals in parentheses.

**Extended Data Table 5:**
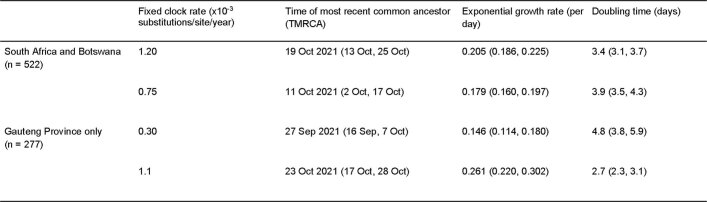
Time of most recent common ancestor, exponential growth rate and doubling time estimates for the full South Africa and Botswana dataset and the reduced dataset of only Gauteng Province genomes under the 4-epoch BDSKY model in which the sampling proportion was allowed to change at 4 equidistantly spaced time points

95% HPD intervals in parentheses.

## Data Availability

All SARS-CoV-2 whole-genome sequences produced by NGS-SA are deposited in the GISAID sequence database and are publicly available subject to the terms and conditions of the GISAID database. The GISAID accession numbers of sequences used in the phylogenetic analysis, including Omicron and global references, are provided in the Supplementary Table 1. Raw reads for our sequences have also been deposited at the NCBI Sequence Read Archive (SRA) (BioProject: PRJNA784038). Other raw data for this study are provided as a supplementary dataset at our GitHub repository (https://github.com/krisp-kwazulu-natal/SARSCoV2_Omicron_Southern_Africa). The reference SARS-CoV-2 genome (MN908947.3) was downloaded from the NCBI database (https://www.ncbi.nlm.nih.gov/). Other publicly available data used in this study are as follows: NCBI SARS-CoV-2 Data Hub (https://www.ncbi.nlm.nih.gov/sars-cov-2/), Protein Data Bank coordinate set 7A94 (https://www.rcsb.org/), Nexstrain global build (https://nextstrain.org/ncov/gisaid/global), Covid-19 Re repository (https://github.com/covid-19-Re), daily Covid-19 case numbers from the Data Science for Social Impact Research Group at the University of Pretoria (https://github.com/dsfsi/covid19za), daily case numbers from OWID (https://github.com/owid/covid-19-data) and the Virus Pathogen Database and Analysis Resource (ViPR) (https://www.viprbrc.org/).

## Code availability

All input files (such as raw data for figures, alignments or XML files), along with all resulting output files and scripts used in the present study are publicly shared at GitHub (https://github.com/krisp-kwazulu-natal/SARSCoV2_Omicron_Southern_Africa).
