# Supplementary material for: Rapid epidemic expansion of the SARS-CoV-2 Omicron variant in southern Africa
Source: Nature. 2022 Jan 7;603(7902):679–86. doi: 10.1038/s41586-022-04411-y (PMC8942855; doi:10.1038/s41586-022-04411-y)
Supplement: Supplementary file 2 — Reporting Summary [file 41586_2022_4411_MOESM2_ESM.pdf]

nature research

Corresponding author(s): Tulio de Oliveira

Last updated by author(s): Jan 6, 2022

Reporting Summary

Nature Research wishes to improve the reproducibility of the work that we publish. This form provides structure for consistency and transparency in reporting. For further information on Nature Research policies, see Authors & Referees and the Editorial Policy Checklist.

Statistics

For all statistical analyses, confirm that the following items are present in the figure legend, table legend, main text, or Methods section.

n/a	Confirmed	
☐	☒	The exact sample size (*n*) for each experimental group/condition, given as a discrete number and unit of measurement
☐	☒	A statement on whether measurements were taken from distinct samples or whether the same sample was measured repeatedly
☐	☒	The statistical test(s) used AND whether they are one- or two-sided<br>*Only common tests should be described solely by name; describe more complex techniques in the Methods section.*
☒	☐	A description of all covariates tested
☐	☒	A description of any assumptions or corrections, such as tests of normality and adjustment for multiple comparisons
☐	☒	A full description of the statistical parameters including central tendency (e.g. means) or other basic estimates (e.g. regression coefficient) AND variation (e.g. standard deviation) or associated estimates of uncertainty (e.g. confidence intervals)
☐	☒	For null hypothesis testing, the test statistic (e.g. *F*, *t*, *r*) with confidence intervals, effect sizes, degrees of freedom and *P* value noted<br>*Give P values as exact values whenever suitable.*
☐	☒	For Bayesian analysis, information on the choice of priors and Markov chain Monte Carlo settings
☐	☒	For hierarchical and complex designs, identification of the appropriate level for tests and full reporting of outcomes
☒	☐	Estimates of effect sizes (e.g. Cohen's *d*, Pearson's *r*), indicating how they were calculated

Our web collection on statistics for biologists contains articles on many of the points above.

Software and code

Policy information about availability of computer code

Data collection	No software was used
Data analysis	Base-calling for GridIon sequencing was performed on MinKNOW software v21.6. Genome assembly was performed with Genome Detective online tool version 1.132 or Exatype NGS SARS-CoV-2 pipeline v1.6.1 or SARSCoV2 RECoVERY (REconstruction of COronaVirus gEnomes & Rapid analYsis) pipeline implemented in the Galaxy instance ARIES (https://aries.iss.it) and validated with Geneious software v.2020.1.2, IG Viewer or Aliview v1.27. Phylogenetic analysis was performed using FastTree2.1, MAFFT v7.490, Nextalign, BEASTv.1.10.4, BEAST2 v2.5.2, and Tracer v.1.7.1. Selection analyses were performed using HyPhy v2.5.33 through the RASCL pipeline. Recombination analyses were performed using 3SEQ, RDP5 and GARD. Lineage classification was performed using the PANGO software suite (lineages v1.2.106). Structure modeling visualization was performed using PyMOL Molecular Graphics System, version 2.2.0. R packages used for data analysis included ggplot, ggtree, seraphim. Custom codes are all available at: https://github.com/krisp-kwazulu-natal/SARSCoV2_Omicron_Southern_Africa.

For manuscripts utilizing custom algorithms or software that are central to the research but not yet described in published literature, software must be made available to editors/reviewers. We strongly encourage code deposition in a community repository (e.g. GitHub). See the Nature Research guidelines for submitting code & software for further information.

Data

Policy information about availability of data

All manuscripts must include a data availability statement. This statement should provide the following information, where applicable:

- Accession codes, unique identifiers, or web links for publicly available datasets
- A list of figures that have associated raw data
- A description of any restrictions on data availability

Data availability Statement: All SARS-CoV-2 whole genome sequences produced by NGS-SA are deposited in the GISAID sequence database and are publicly available subject to the terms and conditions of the GISAID database. The GISAID accession numbers of sequences used in the phylogenetic analysis, including

Omicron and global references, are provided in the Supplementary Table S1. Raw reads for our sequences have also been deposited at the NCBI Sequence Read Archive (SRA) (BioProject accession PRJNA784038). Other raw data for this study are provided as supplementary dataset on our GitHub repository: https://github.com/krisp-kwazulu-natal/SARSCoV2_Omicron_Southern_Africa. The reference SARS-CoV-2 genome (MN908947.3) was downloaded from the NCBI database (https://www.ncbi.nlm.nih.gov/). Other publicly available data used in this study are as follows: NCBI SARS-CoV-2 Data hub (https://www.ncbi.nlm.nih.gov/sars-cov-2/), Protein Data Bank coordinate set 7A94 (https://www.rcsb.org/), Nexstrain global build (https://nextstrain.org/ncov/gisaid/global), Covid-19 Re repository (https://github.com/covid-19-Re), daily Covid-19 case numbers from the Data Science for Social Impact Research Group at the University of Pretoria (https://github.com/dsfsi/covid19za), daily case numbers from OWID (https://github.com/owid/covid-19-data) and the Virus Pathogen Database and Analysis Resource (ViPR) (https://www.viprbrc.org/).

Field-specific reporting

Please select the one below that is the best fit for your research. If you are not sure, read the appropriate sections before making your selection.

☒ Life sciences ☐ Behavioural & social sciences ☐ Ecological, evolutionary & environmental sciences

For a reference copy of the document with all sections, see nature.com/documents/nr-reporting-summary-flat.pdf

Life sciences study design

All studies must disclose on these points even when the disclosure is negative.

Sample size

No sample size calculation was performed; rather all genomic data available at the time of writing for the newly emerged Omicron variant was considered to ensure most accurate analysis and results in a timely manner. At the time of writing (11 December 2021), 553 good quality sequences of the Omicron SARS-CoV-2 variant had been produced by the NGS-SA and Botswana Harvard HIV Reference Laboratory (BHHRL) in South Africa (all fastq in SRA). We believe this was a sufficient sample size as the genomes spanned 8 of the 9 provinces of South Africa, including from multiple districts and two regions of Botswana. For phyloegentic analysis, this was analyzed against a globally representative reference set of SARS-CoV-2 genotypes (n=12 609) spanning the entire genetic diversity observed since the start of the pandemic.

Data exclusions

For phylogenetic analysis and time-calibrated BEAST analysis, genomes were excluded if they presented <90% coverage against the reference AND/OR have sequencing quality problem - e.g. gaps in key regions of the spike protein that causes spurious clustering.

Replication

Reproducibility were performed for maximum likelihood (bootstrap x1000 with FastTree) and bayesian MCMC phylogenetic tree reconstructions. We computed MCMC (Markov chain Monte Carlo) triplicate runs of 100 million states each, sampling every 10,000 steps for the Omicron dataset. All attempts at replication were successful and the MCC tree for the Omicron cluster was of high support.

Randomization

Experimental groups consisted of weekly batches of residual patient nasopharyngeal swabs selected for sequencing to determine the progression of weekly lineage prevalence as part of surveillance. Samples for weekly SARS-CoV-2 sequencing in South Africa and Botswana were selected at random from all relevant divisions in each country, without any clinical or geographical bias. Generally, part of the Network for Genomic Surveillance in South Africa (NGS-SA), five sequencing hubs receive randomly selected samples for sequencing every week according to approved protocols at each site. In response to a rapid resurgence of COVID-19 in Gauteng Province in November, we enriched our routine sampling with additional samples from those areas.

Blinding

Geographical blinding of data was not necessary for the study as it involves phylogeographical analysis. Other types of blinding were also not necessary as this was not a cohort study.

Reporting for specific materials, systems and methods

We require information from authors about some types of materials, experimental systems and methods used in many studies. Here, indicate whether each material, system or method listed is relevant to your study. If you are not sure if a list item applies to your research, read the appropriate section before selecting a response.

Materials & experimental systems

n/a	Involved in the study	
☒	☐	Antibodies
☒	☐	Eukaryotic cell lines
☒	☐	Palaeontology
☒	☐	Animals and other organisms
☐	☒	Human research participants
☒	☐	Clinical data

Methods

n/a	Involved in the study	
☒	☐	ChIP-seq
☒	☐	Flow cytometry
☒	☐	MRI-based neuroimaging

Human research participants

Policy information about studies involving human research participants

Population characteristics

We obtained samples consisting of remnant nucleic acid extracts or remnant nasopharyngeal and oropharyngeal swab samples from routine diagnostic SARS-CoV-2 PCR testing from public and private laboratories in South Africa. The Omicron genomes in

this study came from patients of ages 0-82, with an approximately equal distribution of males and females, for which the Omicron genotype was confirmed by sequencing.

Recruitment	As part of the Network for Genomic Surveillance in South Africa (NGS-SA), five sequencing hubs receive randomly selected samples for sequencing every week according to approved protocols at each site. In response to a rapid resurgence of COVID-19 in the province of Gauteng in November, we enriched our routine sampling with additional samples from this area. One bias that may be present is the ability to sequence only from the pool of patients that seek testing and that receive a positive PCR test.
Ethics oversight	The genomic surveillance in South Africa was approved by the University of KwaZulu–Natal Biomedical Research Ethics Committee (BREC/00001510/2020), the University of the Witwatersrand Human Research Ethics Committee (HREC) (M180832), Stellenbosch University HREC (N20/04/008_COVID-19), University of Cape Town HREC (383/2020), University of Pretoria HREC (H101/17) and the University of the Free State Health Sciences Research Ethics Committee (UFS-HSD2020/1860/2710). The genomic sequencing in Botswana was conducted as part of the national vaccine roll-out plan and was approved by the Health Research and Development Committee (Health Research Ethics body, HRDC#00948 and HRDC#00904). Individual participant consent was not required for the genomic surveillance. This requirement was waived by the Research Ethics Committees.

Note that full information on the approval of the study protocol must also be provided in the manuscript.
